# Deep learning trained on hematoxylin and eosin tumor region of Interest predicts HER2 status and trastuzumab treatment response in HER2+ breast cancer

**DOI:** 10.1038/s41379-021-00911-w

**Published:** 2021-09-07

**Authors:** Saman Farahmand, Aileen I. Fernandez, Fahad Shabbir Ahmed, David L. Rimm, Jeffrey H. Chuang, Emily Reisenbichler, Kourosh Zarringhalam

**Affiliations:** 1 University of Massachusetts-Boston, Department of Mathematics, Boston, MA, USA.; 2 University of Massachusetts-Boston, Computational Sciences PhD Program, Boston, MA, USA.; 3 Yale University, Yale School of Medicine, Department of Pathology, New Haven, CT, USA.; 4 The Jackson Laboratory for Genomic Medicine, Farmington, CT, USA.; 5 UCONN Health, Department of Genetics and Genome Sciences, Farmington, CT, USA.

## Abstract

The current standard of care for many patients with HER2-positive breast cancer is neoadjuvant chemotherapy in combination with anti-HER2 agents, based on HER2 amplification as detected by in situ hybridization (ISH) or protein immunohistochemistry (IHC). However, hematoxylin & eosin (H&E) tumor stains are more commonly available, and accurate prediction of HER2 status and anti-HER2 treatment response from H&E would reduce costs and increase the speed of treatment selection. Computational algorithms for H&E have been effective in predicting a variety of cancer features and clinical outcomes, including moderate success in predicting HER2 status. In this work, we present a novel convolutional neural network (CNN) approach able to predict HER2 status with increased accuracy over prior methods. We trained a CNN classifier on 188 H&E whole slide images (WSIs) manually annotated for tumor Regions of interest (ROIs) by our pathology team. Our classifier achieved an area under the curve (AUC) of 0.90 in cross-validation of slide-level HER2 status and 0.81 on an independent TCGA test set. Within slides, we observed strong agreement between pathologist annotated ROIs and blinded computational predictions of tumor regions / HER2 status. Moreover, we trained our classifier on pre-treatment samples from 187 HER2+ patients that subsequently received trastuzumab therapy. Our classifier achieved an AUC of 0.80 in a five-fold cross validation. Our work provides an H&E-based algorithm that can predict HER2 status and trastuzumab response in breast cancer at an accuracy that may benefit clinical evaluations.

## INTRODUCTION

Human epidermal growth factor 2 (HER2) is a proto-oncogene that is amplified in 15–20% of breast cancer cases^1^. In the absence of systemic adjuvant therapy, HER2 gene amplification or protein overexpression is associated with aggressive clinical behavior and poor survival outcome^2,3^. Fortunately, anti-HER2 treatments such as trastuzumab significantly improve survival outcome^4^. Response and overall survival rates of trastuzumab treatment, in combination with chemotherapy, for HER2+ cases for metastatic breast cancer range from 10–41% and 56–85% respectively, while the response and survival rates for non-metastatic cases range from 50–70% and 56–88%^5-14^. As a result, HER2 testing is routinely applied in invasive breast cancer cases and used as the sole biomarker for anti-HER2 treatment^15,16^. However not all clinically defined HER2+ cases respond to treatment nor do tumors lacking HER2 amplification^16^. Current ASCO/CAP^16^ standards for determining HER2 gene amplification and protein overexpression are in situ hybridization (ISH) and immunohistochemistry (IHC) respectively^16-18^, though discordance between ISH and IHC is not uncommon and can lead to HER2+ overdiagnosis. One solution may be hematoxylin & eosin (H&E) images, which are commonly generated during pathological analysis and widely abundant, providing opportunities for novel data-driven computational methods. Machine learning-based predictors trained on annotated H&E data could be a potent technology to improve the speed, accuracy, and cost of predicting HER2 status and anti-HER2 treatment response.

In recent years, there has been a growth in machine learning approaches, especially deep learning, in the field of pathology^19^. These typically utilize Convolutional Neural Network (CNN) architectures, such as AlexNet^20^, GoogleNet^21^, or ResNet^22^, etc., pre-trained on generic images, and then fine-tune them by retraining the last layers for a specific classification task. This approach is typically referred to as “transfer-learning”. In contrast, the CNN models can be trained using a “full-training” strategy, where no pre-training is utilized, and all CNN parameters are trained using the training dataset of interest. Representative examples of CNN-based models for pathology applications include tumor/benign classification^23-26^, predicting mutations in key genes^23,24,27,28^, cancer subtype classification and morphology analysis^23,29^, and treatment outcome prediction^30,31^. These models have shown impressive performance, demonstrating that subtle molecular features of cancer may be discernible from H&E images.

The objective of this work is to provide a deep learning framework to predict HER2 status and response to trastuzumab therapy from breast cancer H&E slides. Recent studies have addressed aspects of this problem with moderate success^32-34^. In this work, we trained a HER2 status predictor model on 188 HER2± H&E slides generated from the Yale Pathology electronic database (Yale HER2 cohort) and utilized 187 HER2± H&E slides from The Cancer Genomic Atlas (TCGA) BRCA cohort as an independent test set. For trastuzumab response prediction, we used a cohort of 85 pre-treatment HER2+ samples from the Yale Pathology electronic database (Yale Response cohort). In our approach, we employed both transfer and full training strategies. Importantly, we utilized tiles from H&E Whole Slide Images (WSIs), manually annotated for ROIs by our pathology team.

**Fig. 1:**
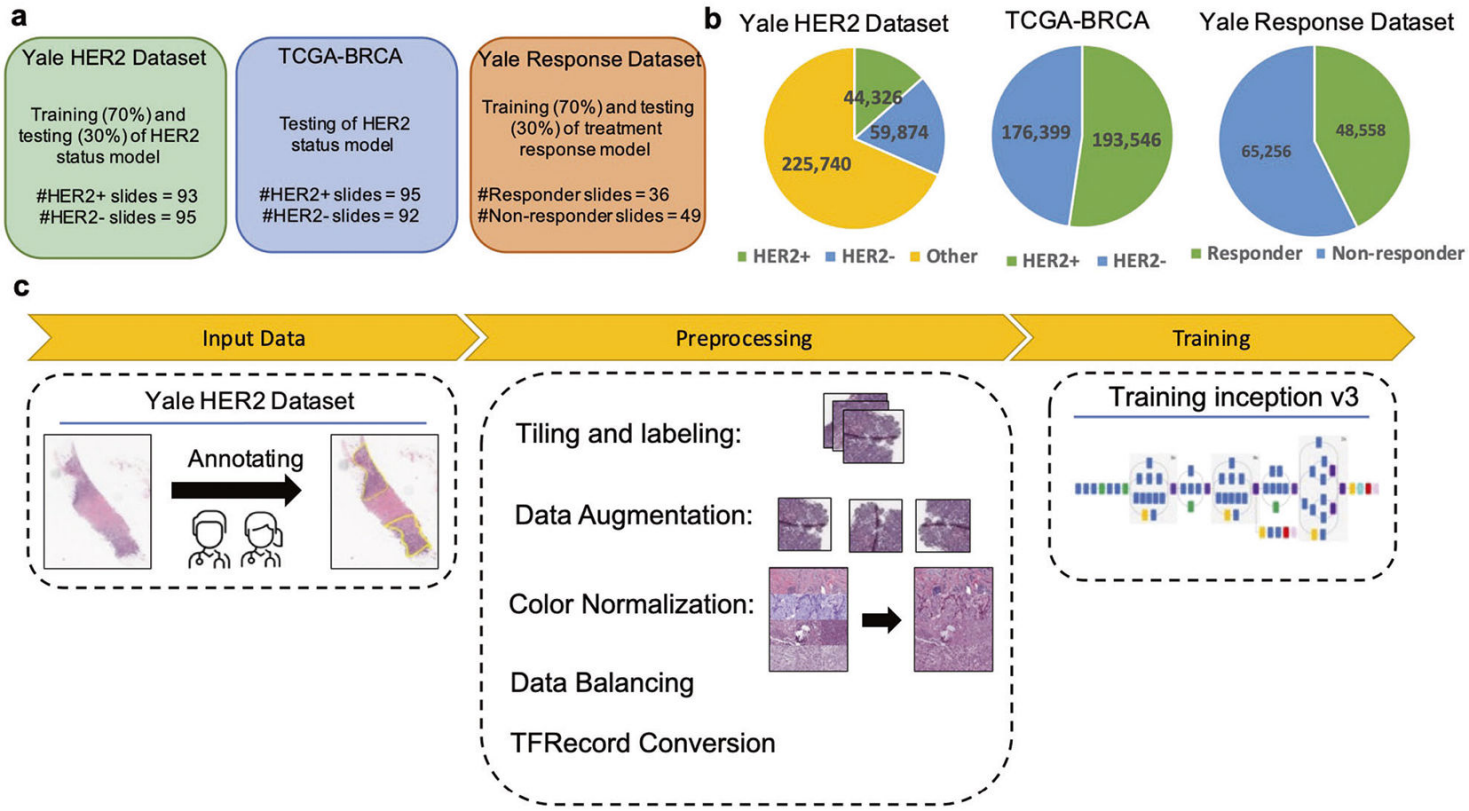
Datasets and study design for HER2 status and Trastuzumab response classification. **a** Datasets generated and used for training and testing the models. **b** Number of tiles in each class. The whole TCGA-BRCA slides as an independent test set were used for testing (we only showed proportion of tiles corresponding to only tumor regions here). For the response model, we only used the tumor regions to train and test the model which the proportion of tiles in each class are depicted here. **c** The main steps for preprocessing of slides and training the model. Our pathology team performed quality checks and annotated the ROIs in every slide to distinguish HER2+ tumor regions, HER2− tumor regions, and other non-tumor regions. In the preprocessing step, slides were tiled into 512 × 512 pixel windows, and background tiles were removed. Data were randomly split into 70% for training and 30% for testing for both Yale cohorts. The TCGA-BRCA cohort was used to independently validate the HER2 status prediction model. Data augmentation and color normalization were utilized to increase reproducibility. Classes were balanced with down- and up- sampling. Tiles were randomly sorted and converted into TFRecords to train the inception v3-based model. Test data was used to assess model performance. Predictions were visualized on WSIs with heatmaps.

We demonstrate that the use of tile-level annotations significantly improves classification accuracy compared to previous approaches for both HER2 status and trastuzumab response. Figure 1 shows an overview of our approach.

## MATERIALS AND METHODS

### Data and study design

#### Approval of human tissue data.

All tissues and data were retrieved under permission from the Yale Human Investigation Committee protocol #9505008219 to DLR

#### Yale HER2 cohort.

188 HER2 positive and negative invasive breast carcinomas were identified by retrospective search of the Yale Pathology electronic database with HER2 positive cases defined as those with 3+ score by immunohistochemistry (IHC) as defined by American Society of Clinical Oncology/College of American Pathologists (ASCO/CAP) clinical practice guidelines^18^. The samples were reviewed prior to scanning to ensure they were strong complete membranous staining. These were selected for the training component to avoid any ambiguity. H&E slides generated at Yale School of Medicine include 93 HER2+ and 95 HER2− slides. The slides were scanned at Yale Pathology Tissue Services and underwent a slide quality check before they went into the scanner. Broken slides, slides with broken coverslips, and slides with no/minimal tissue were removed. The tissue samples were scanned using Aperio ScanScope Console (v10.2.0.2352) using bright field whole slides scanning at 20× magnification.

#### TCGA HER2 cohort.

A total of 668 TCGA-BRCA HER2± samples with available HER2 status were downloaded from the GDC portal. Slides were visually inspected by our pathology team to exclude low-quality samples with tissue folding or those that appeared to be from frozen tissue. A total of 187 samples (92 HER2− and 95 HER2+) were retained for use as independent test set.

#### Yale trastuzumab response cohort.

The response cohort cases were identified also by retrospective search of the Yale Pathology electronic database. Cases included those patients with a pre-treatment breast core biopsy with HER2 positive invasive breast carcinoma who then received neoadjuvant targeted therapy with trastuzumab± pertuzumab prior to definitive surgery. HER2 positivity was defined as previously described for the HER2 negative/positive cohort. The response to targeted therapy was obtained from the pathology reports of the surgical resection specimens and dichotomized into responders or non-responders. Those with a complete pathologic response, defined as no residual invasive, lymphovascular invasion or metastatic carcinoma, were designated as responders (*n* = 36). Cases with only residual in situ carcinoma were included in the responder category. Those cases with any amount of residual invasive carcinoma, lymphovascular invasion or metastatic carcinoma were categorized as non-responders (*n* = 49).

### Data preparation

#### Data annotation.

Annotation of digital slides was performed, circling areas of invasive carcinoma ROIs. Regions of necrosis, in situ carcinoma or benign stroma and epithelium were excluded. The images were annotated with ROIs associated to HER2± tumor area (TA) by a senior breast pathologist. The annotations were marked tumor boundaries and annotated by Aperio ImageScope software^35^. The annotations were exported from the Aperio software in The Extensible Markup Language (XML) format, including X and Y coordinates corresponding to the annotated regions. We used these coordinates for each slide image to tile these regions separately from the rest of the image, labeled as HER2+ or HER2− class.

#### Data prep-processing.

Yale cohort slides were randomly split and assigned to 70% for training and 30% for testing. Image slides were tiled into non-overlapping patches of 512 × 512 pixels in 20× magnification. Regions with excess background or containing no tissue as well as regions excess fat were removed as previously described^24^. Tiles were shuffled and assigned to Tensorflow Records (TFRecords). To mitigate the effects of class imbalance, we utilized undersampling of the majority class for two-class classification and undersampling and oversampling of the minority and majority classes respectively for three-class classification.

#### Data augmentation and normalization.

Data augmentation was performed on training tiles with 90-, 180-, and 270-degree rotations and as well as horizontal and vertical flips. To standardize the color space and address potential batch-effects, we utilized a deep learning-based generative model to normalize the stain color across training and independent test data sets^36^. The normalization method is fully unsupervised and does not utilize class label information in the normalization process.

### Model training and assessment

#### CNN architecture and training.

We utilized an Inception v3 architecture^36^ to predict HER2 status in breast cancer and trastuzumab treatment in HER2+ samples. Models were trained using both transfer learning and full training strategies. Transfer learning model parameters were set according to optimal values from the ImageNet competition^37^. The parameters of the last layer of the network were fine-tuned on samples using back propagation. To quantify the impact of ROIs on training, we utilized three different training schemes to predict the HER2 status. **1**. *Unannotated two-way classifier*: Tiles were assigned the label according to the WSI (Positive or Negative) and ROIs were not taken into consideration in training. **2**. *Annotated two-way classifier*: Only tiles falling within the ROIs were utilized in training. Exterior regions (including stromal cells, necrotic cells and/or mixed of tumor and normal cells) were not taken into consideration for training and within ROI tiles were assigned the WSI label. **3**. *Annotated three-way classifier*: Both within ROIs and exterior regions were utilized to train a multi-way classifier. Within ROI tiles were labeled as Positive or Negative according to the WSI label. Tiles in the exterior regions were labeled as “Other” independent of the WSI label. A similar strategy was taken to train a binary classifier for the trastuzumab response predictor. A softmax link function was utilized as loss and predicted probabilities were calculated for each tile. We used RMSProp69 optimization with learning rate of 0.1, weight decay of 0.9, momentum of 0.9, and epsilon of 1.0 to train the model (Fig. 1).

#### Model assessment.

Model performance was evaluated on test tiles. Slide-level probabilities were calculated by averaging the output probabilities for HER2+ and HER2− classes and the final slide-level label was decided using a 0.5 cutoff threshold on the aggregate probabilities. Model performance was assessed on a per-tile and a per-slide basis. The ROC curves and the corresponding AUC were calculated, and 95% Confidence Intervals (CIs) were estimated by 1000 iterations of the bootstrap method^38^. For the treatment response predictor, we also utilized a 5-fold cross validation, which was possible due to the smaller number of samples. The mean and standard deviation of AUC values were calculated using prediction on each fold.

#### Computational configuration.

All analyses were performed in Python. Inception V3 code was adopted from^24^. Images were analyzed and processed using OpenSlide. Classification metrics were calculated using the Scikit-learn package^39^. All of the computational tasks were performed on Massachusetts Green High Performance Computing Cluster (MGHPCC) on nodes with the following specification: 8 CPUs with 64 GB RAM, Tesla V100 GPUs with 256 GB RAM. TensorFlow and TF-slim documentations and NVIDIA GPUs support were followed to setup and configure CUDA 8.0 Toolkit and cuDNN v5.1.

## RESULTS

### HER2 status classification using unannotated slides

**Fig. 2:**
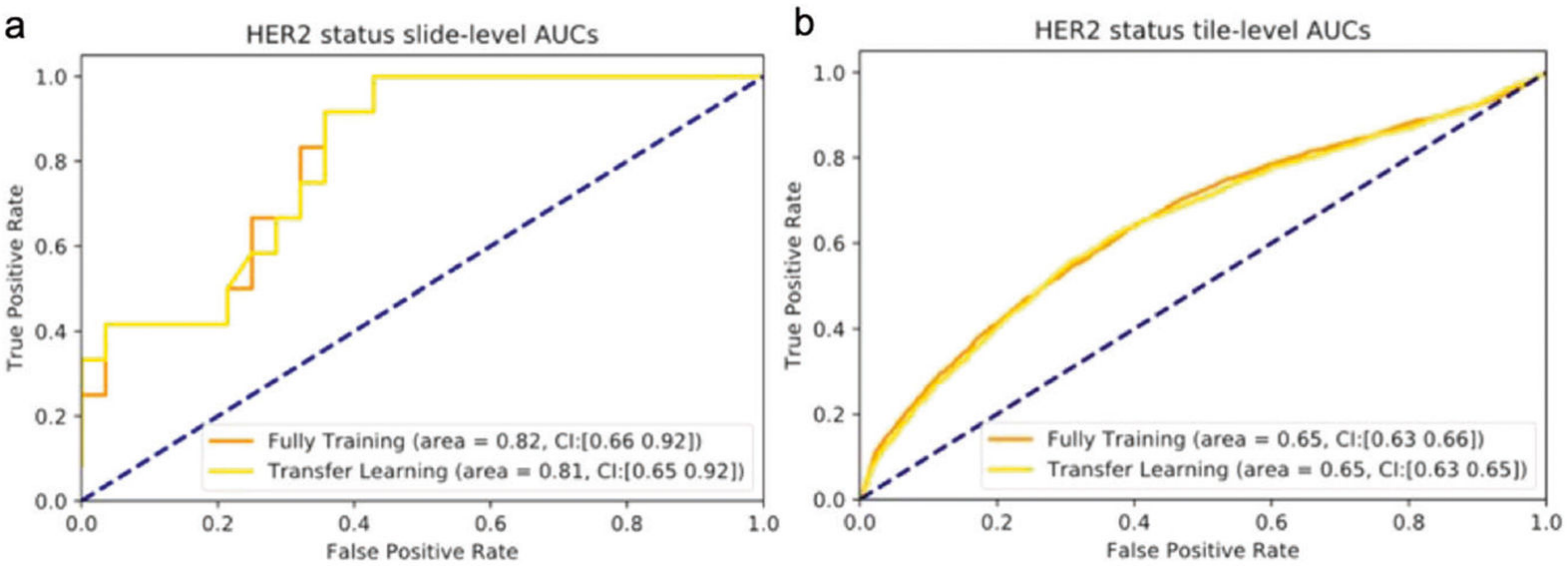
HER2 status classification using unannotated slides. AUC/ROC for HER2 status classification at the slide-level (**a**) and at the tile-level (**b**) for both transfer learning and the fully trained models.

As a base model and for benchmarking with previous approaches, we trained a CNN model to predict HER2 status using unannotated slides from the Yale HER2 cohort (93 HER2+ and 95 HER2−). In this classification scheme (two-way unannotated, Methods) WSIs were tiled to non-overlapping regions and each tile was assigned the label of its corresponding slide (HER2+ or HER2−). The CNN model was trained using both transfer learning as well as full training. Prediction on the left-out data showed a slide-level AUC 0.81 (95% CI, 0.65–0.92) in transfer learning and AUC 0.82 (95% CI, 0.66–0.92) in the fully trained model. At the tile-level the model achieved an AUC of 0.65 (95% CI, 0.63–0.66) in transfer learning and 0.65 (95% CI, 0.63–0.65) in the fully trained model (Fig. 2). In the following sections, we will present classification schemes that improve on these widely used two-way classifiers, resulting in significant gains in model accuracy and generalizability.

### HER2 status classification using annotated slides

**Fig. 3:**
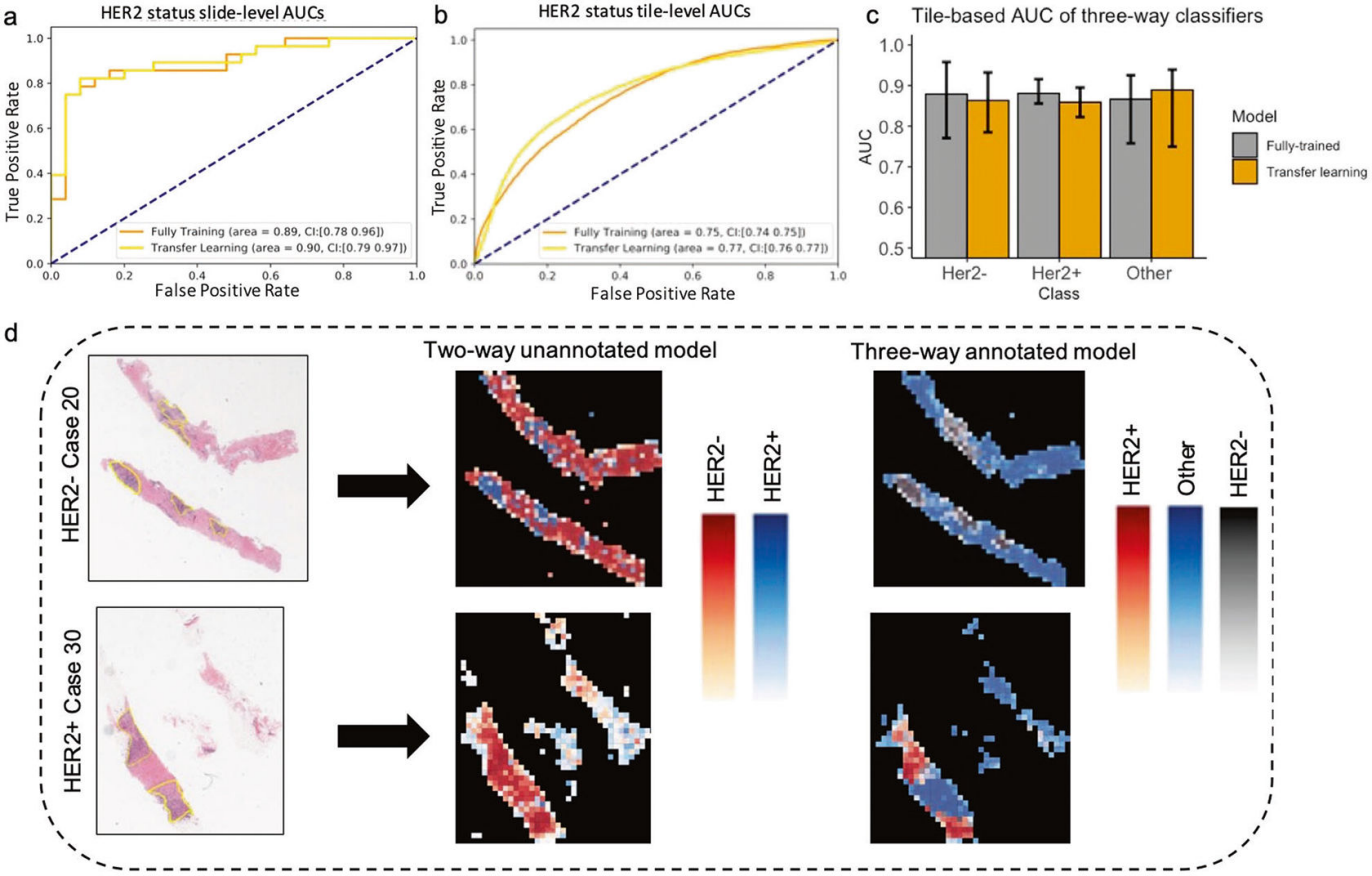
HER2 tumor status classification using annotated slides. AUC/ROC for HER2 status classification at the slide-level (**a**) and at the tile-level (**b**) for both transfer learning and the fully trained models. **c** Tile-level AUC of the three-way classifiers. **d** Two representative H&E slides from the test set with corresponding heatmaps based on predicted probabilities by the CNN model. Yellow regions indicate the ROIs determined by our pathology team. The middle panel shows predicted heatmaps for unannotated model and the right panel shows the predicted heatmaps from three-way annotated model.

We hypothesized that using tiles from ROIs may reduce irrelevant features and enable the CNN to better learn features specific to HER2± tumor status. Our pathology team annotated the Yale HER2 cohort to mark the regions corresponding to the invasive tumor cells while excluding regions such as necrosis, in situ carcinoma, benign stroma and epithelium. Each slide was masked according to these manual annotations, then broken into tiles for analysis with each tile categorized as tumor or Other. The tumor tiles were categorized as either HER2+ or HER2−, yielding 3 classes for training: HER2+, HER2− and Other. To draw direct comparison with the previous classifier, we trained another two-way classifier, this time trained on HER2± tiles only (two-way annotated, Methods). Figure 3a, b presents the AUC values of the CNN classifier for the two-way annotated model using both full training and transfer learning approaches. The model achieved a slide-level AUC of 0.90 (95% CI, 0.79–0.97) and a tile-level AUC of 0.77 (95% CI, 0.76–0.77) in the transfer learning approach, and AUC of 0.89 (95% CI, 0.78–0.96) and a tile-level AUC of 0.75 (95% CI, 0.74–0.75) in the fully trained model. We generated a heatmap using tile-predicted probabilities to visualize the predictions made by the model (Fig. 3d middle column). Although the prediction performance at the slide-level is high, the tile-level heatmaps do not show the same level of performance compared with tile-level pathologist annotations (Fig. 3d first column yellow regions).

Next, we tested whether including tiles from exterior regions of ROIs can improve the tile-level accuracies and ROI visualizations by heatmaps. Our rationale for including these tiles was that the classifier trained on HER2± tiles is likely unable to predict class label of the exterior tiles. We trained a three-way classifier on HER2+, HER2−, and Other tiles (annotated three-way, Methods). Figure 3c shows the tile-level AUCs for models trained using transfer learning or full training. The fully trained CNN model predicted the HER2− status with an AUC of 0.88 (95% CI, 0.77–0.95), and HER2+ with an AUC of 0.88 (95% CI, 0.85–0.91) and Other class with an AUC of 0.87 (95% CI, 0.75–0.92). The transfer learning model achieved similar AUCs. This is a clear increase in the CNN’s tile-level AUC compared with the two-way annotated classifier (Fig. 3b), indicating that features from non-HER2± tiles can decrease the confusion between HER2+ and HER2− tiles. Figure 3d right column illustrates the heatmaps produced by the three-way classifier. There is strong agreement between the heatmap from the three-way classifier and pathologist annotated ROIs, indicating the utility of our model for automatic ROI detection.

### Model validation on independent test set

We next validated the HER2 status classifier on an external independent test set. For this analysis, we downloaded a dataset consisting of 569 HER2− and 99 HER2+ WSIs of H&E stained sections of formalin fixed paraffin-embedded (FFPE) samples from TCGA-BRCA cohort. Our pathology team performed quality control to exclude samples with poor scanning and staining quality, resulting in 197 samples being excluded from further analysis. Slides were processed and tiled as in the Yale HER2 cohort, resulting in 176399 HER2+. and 193546 HER2− tiles. Since the training and test cohorts were from independent sources, we performed a stain-color normalization step using a deep generative model^36^ to scale the TCGA cohort to the Yale cohort (Methods). The CNN HER2 status classifier, trained on the Yale HER2 cohort was used to make predictions on the TCGA cohort. The AUCs of the model performance are 0.81 (95% CI: 0.73–0.84) at the slide-level and 0.65 (95% CI: 0.54–0.69) at the tile-level.

**Fig. 4:**
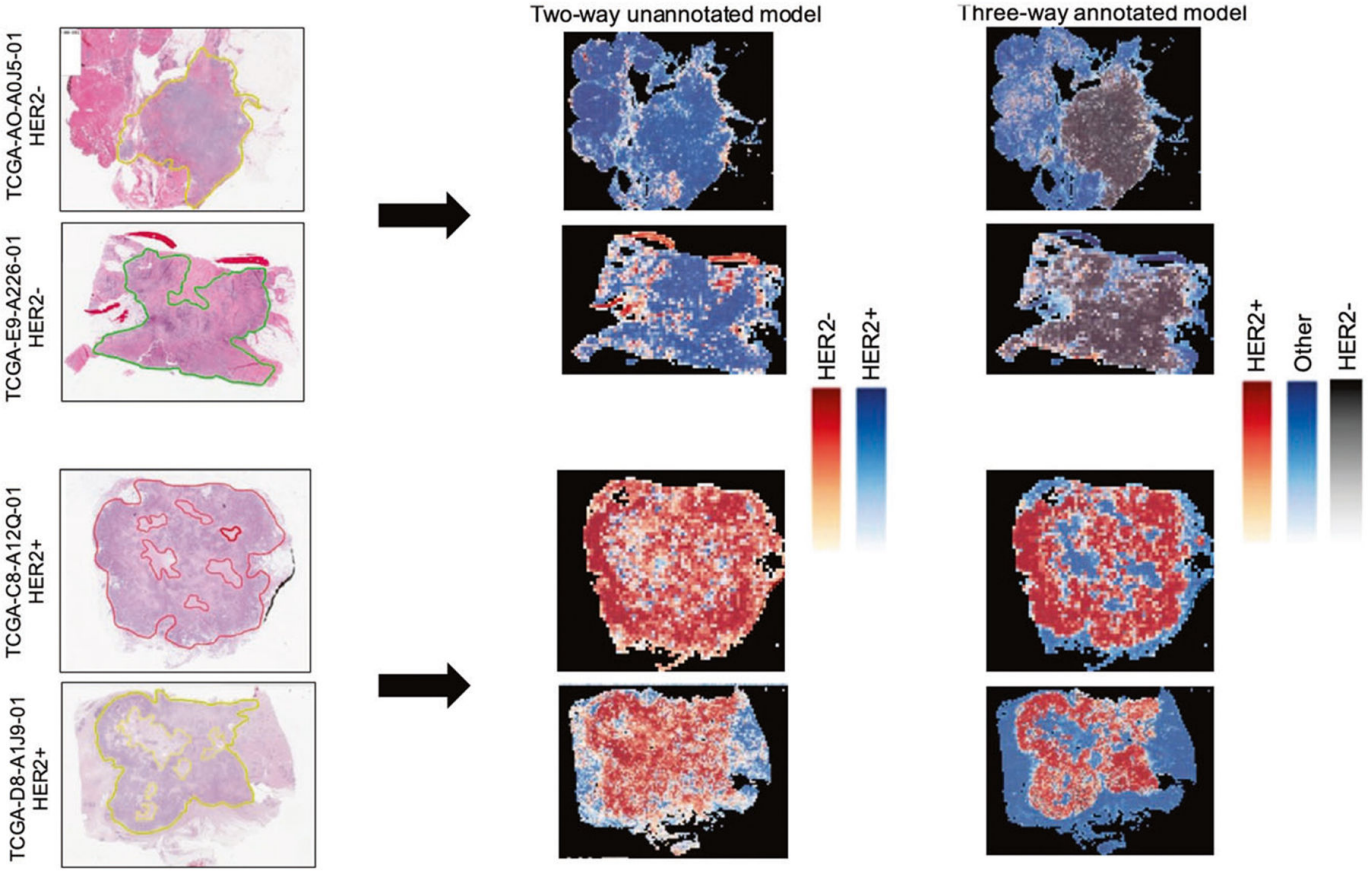
Representative H&E slides from TCGA test set and their predicted heatmaps. Left column slides along with indicated ROIs. Middle panel: Two-way unannotated classifier heatmaps. Right panel: Three-way annotated classifier heatmaps.

We also tested whether ROIs can be accurately detected in the test set. As in the Yale HER2 cohort, our pathology team annotated the TCGA-BRCA cohorts to mark ROIs. Figure 4 shows two representative samples of annotations (yellow regions), heatmaps predicted by the unannotated two-way classifier, and the heatmap produced by the annotated three-way classifier. There is a high-level of agreement between the ROIs and the predictions made by the three-way classifier, demonstrating the generalizability of our automatic ROI detection.

### Deep learning predicts trastuzumab treatment outcome

**Fig. 5:**
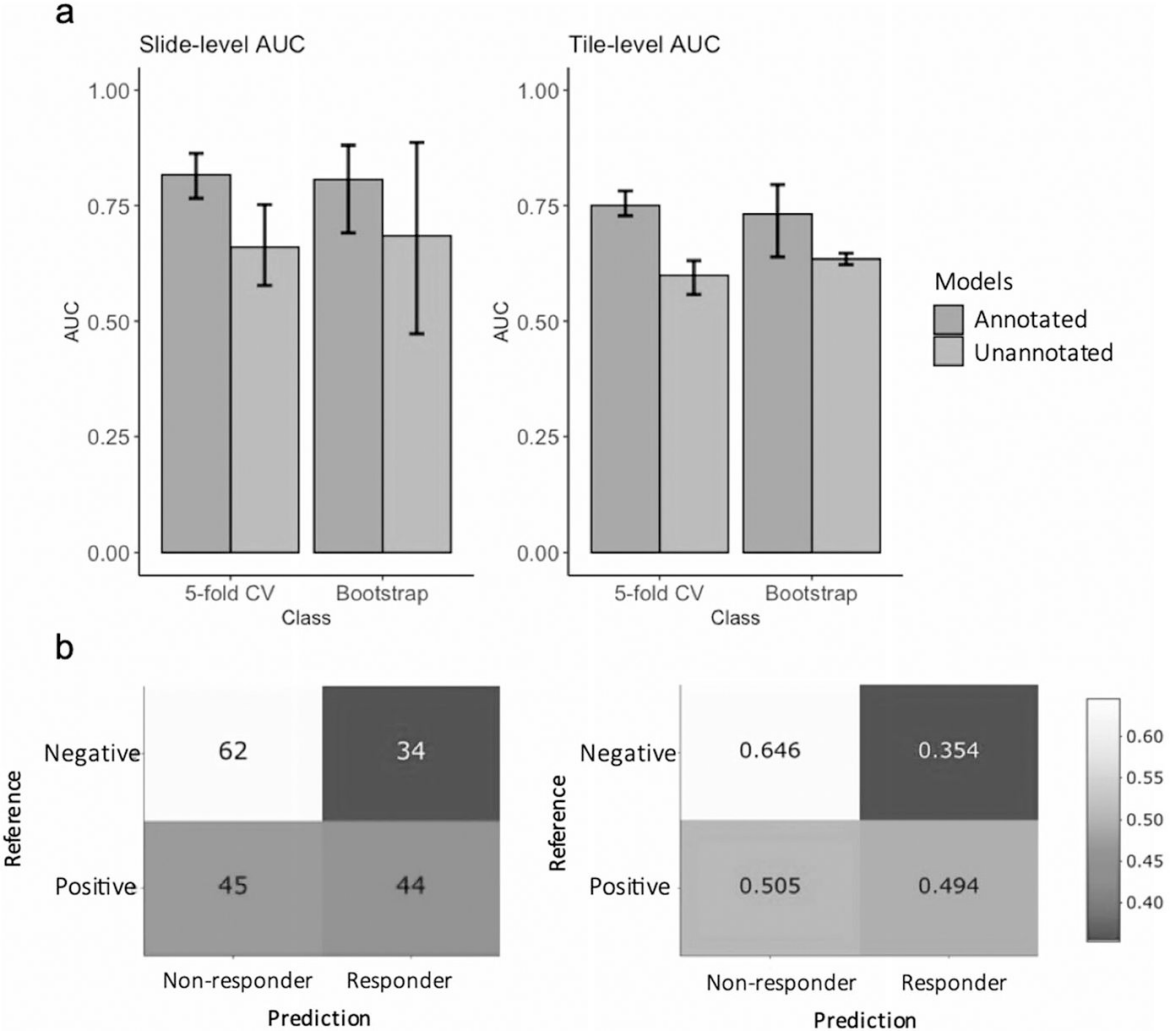
Trastuzumab response prediction. **a** Slide-level and Tile-level AUC/ROC for both annotated and unannotated models using bootstrapping and 5-fold cross validation. **b** Confusion matrix of the HER2 status classifier used as Trastuzumab response predictor.

We next tested whether deep learning trained on H&E slides from HER2+ patients can predict trastuzumab treatment outcome. For this study, we utilized pre-treatment H&E slides from the Yale trastuzumab cohort. As with the previous samples, our pathology team annotated the slides to mark the invasive tumor cells area. In addition to model assessment using test sets and CI estimation with bootstrapping, we performed a 5-fold cross validation to more stringently assess model performance. The unannotated model achieved an AUC of 0.68 (95% CI: 0.47–0.88) at the slide-level and an AUC of 0.63 (95% CI: 0.62–0.65) at the tile-level. On the other hand, the annotated models achieved an AUC of 0.80 (95% CI: 0.69–0.88) at the slide-level and an AUC of 0.73 (95% CI: 0.63–0.79) at the tile level (Fig. 5). As in the HER2 status classifier, the improvement in AUCs shows the importance of annotations in training of deep learning classifiers for response prediction.

We also tested whether the HER2 status classifier can directly predict trastuzumab treatment response. This is important as HER2 status is the clinical biomarker for anti-HER2 treatment. For this test, the Yale HER2 response data was used as input to the HER2 status CNN-classifier, and the predictions made by the classifier (HER2±) were tabulated against the response labels. Figure 5b shows the confusion matrix. As shown, 50% of HER2+ samples were predicted as responders, while 65% of the HER2− samples were predicted as non-responders. These results demonstrate that although HER2 status, as determined by traditional IHC/ISH methods, can moderately predict trastuzumab response, more specifically trained models are needed to better identify patients who would benefit from trastuzumab treatment therapy. Taken together, these results support the feasibility of image-based biomarkers for prediction of trastuzumab therapy and the ability of the deep learning model to identify morphological variations associated with treatment outcome. Trastuzumab response predictors, such as the one presented in this study, have the potential to augment HER2 status testing for treatment recommendation in HER2+ patients.

## DISCUSSION

In this work, we presented CNN-based classifiers for determining HER2 status and trastuzumab response prediction. Using high-quality slides carefully annotated by expert pathologists, we were able to reduce the feature space and hence the number of required samples for training our classifiers. To minimize heterogeneity and avoid confusion with borderline cases during training, we only utilized ROIs from the Yale cohort cases with IHC score 3+ to train the model. The testing was performed on all TCGA samples that passed the quality control (Methods). A two-way classifier of HER2 status, trained on within-tumor ROI tiles achieved a slide-level AUC of 0.90 in cross-validation and 0.81 on an independent test set. To increase the tile-level accuracy and the predicted ROI heatmaps, we devised a three-way classification scheme and trained a multi-way classifier using tiles from within as well as the exterior ROI regions. The three-way classifier was able to distinguish tiles from each class with high accuracy (AUCs: HER2+ 0.88, HER2− 0.88, Other: 0.87). Heatmaps produced by the three-way classifier show a remarkable agreement with pathology annotations, both in the slides from the training set as well as the slides from the independent set.

Three recent studies have also addressed aspects of this problem. Bychkov et al.^34^, investigated whether predicting HER2 status using a CNN model can guide the choice of therapy. The study utilized cancer tissue samples from FinProg patient series^40^, the FinProg validation series^41^, and the FinHer clinical trial^42^, all of which had HER2 amplification determined by CISH. Their CNN model, trained on random tile crops of size 950 × 950 from 693 H&E-stained patient samples from the FinProg series was able to predict tile-level HER2 status with AUC 0.70 (95% CI, 0.63–0.77) in a 5-fold cross validation and AUC 0.67 (95% CI, 0.62–0.71) on 712 test images from the FinHer dataset. They did not report slide-level AUCs. In their approaches, only tiles from the center crop (2100 × 2100 pixels) of the WSI were used to test the prediction performance, whereas in our approach tile-level AUC was estimated using all test tiles. As such, direct comparison between the methods is confounded by their test-tile selection procedure. On the other hand, our ensemble procedure for slide-level HER2 status prediction results in a significant increase in AUCs, demonstrating the robustness and the generalizability of our approach. They also devised a score for HER2 status (H&E-*ERBB2* score) and reported that CISH HER2+ patients with high H&E-*ERBB2* score treated with trastuzumab had a more favorable distant disease-free survival rate than those with a low H&E-*ERBB2* score (Hazard Ratio, 0.37; 95% CI, 0.15–0.93; *P* = 0.034). CISH HER2+/ high H&E-*ERBB2*-positive patients not treated with trastuzumab also exhibited less favorable disease-free survival (Hazard ratio, 2.03; 95% CI, 0.69–5.94; *P* = 0.20). These findings indicate that an H&E-based score can contribute to a more accurate prediction of trastuzumab efficacy than CISH alone, but at the same time it is critical to further improve on these AUC values to optimize applicability to clinical practice.

In another related study Rawat et al.^33^ trained a patch-based CNN classifier on 939 TCGA H&E images with patch-sizes of 224 × 224. Their model achieved a slide-level HER2 AUC of 0.71 (TCGA, *n* = 124) in a 5-fold cross validation. They also tested the generalizability of their model using an independent cohort from The Australian Breast Cancer Tissue Bank (ABCTB)^40^. Their model achieved a slide-level Her2 AUC = 0.79 (ABCTB, *n* = 2487). Interestingly, this AUC is larger than their within-TCGA cross-validation AUC (0.71), on which their model was trained, presumably due to the higher quality of ABCTB slides. In both cases, our cross-validation and independent test AUC on TCGA cohort (0.9, and 0.81) improves upon these results.

Finally, Naik et al.^32^, developed a ReceptorNet ER+/ER− binary classifier trained on patches sampled from 2535 H&E WSIs from Australian Breast Cancer Tissue Bank (ABCTB) and 1014 H&E WSIs from 939 patients from TCGA with ER, PR, and HER2 status determined by pathologists using IHC. Their classifier achieved an Area Under the Curve (AUC) of 0.899 (95% CI: 0.884–0.913) on cross-validation and an AUC of 0.92 (95% CI: 0.892–0.946) on the test set. They reported that the ER+/ER− classifier performed significantly better on HER2− samples (AUC = 0.927, 95% CI: 0.912–0.943) as compared to HER2 samples (AUC = 0.768, 95% CI: 0.719–0.813). Additionally, they trained and evaluated their classifier to predict PR and HER2 status and obtained an AUC of 0.810 (95% CI: 0.769–0.846) on PR and an AUC of 0.778 (95% CI: 0.730–0.825) on HER2.

A key improvement of our method compared to these previous approaches is our use of tumor Regions of Interest (ROI) annotations during training. These annotations allowed us to train and evaluate the three-way classification model for HER2+, HER2−, and non-tumor tiles within each WSI. In contrast, previous approaches have utilized a weakly supervised two-way (HER2+/HER2−) classification model based on slide-level rather than tile level annotations. On the other hand, reliance of our models on manually annotated ROIs make the approach less generalizable to other cancer types where such detailed annotations are not readily available. This is a potential drawback of our approach, however, tissue imaging is rapidly progressing in scale and we anticipate that annotated training datasets as well as increasingly accurate computational tumor ROI predictors will become more prevalent.

Another strength of our method is the use of deep learning-based color normalization^36^ to remove batch-effects and improve generalizability to independent datasets. We utilized a deep learning-based color normalization scheme developed by Zanjani et al.^36^. Color and intensity variations between H&E samples from different medical centers or even within the same laboratory samples generated at various trials or time periods is common^43^. Variations in specimen sample preparation protocol, staining protocols, scanning, and imaging device characteristics are some of the contributing factors. As such, H&E stain color normalization has been studied and used in deep learning approaches^44-46^. Recently^43^, Howard et al. showed that features extracted by deep learning models trained on H&E images vary substantially across data sets. They point out that color normalization alone may not be sufficient to address confounding factors and generalizability of deep learning models to independent datasets remains a challenging task. However, this may be limited to more subtle molecular features of cancer. In our case, color normalization resulted in a small increase in model accuracy, and further investigation of similar effect are likely important to understanding the variations in predictive accuracy across different cohorts.

Taken together, the significant improvement in slide-level and tile-level AUCs relative to those from our unannotated model and previous results^32-34^ indicate the importance of using pathology annotation to guide targeted feature learning.

Although the response rate to trastuzumab therapy in HER2+ patients has been good, augmenting HER2 status determination with more accurate methodologies for treatment response prediction has the potential to improve patient care. Using pre-treatment samples from HER2+ positive patients with known trastuzumab response, we trained a classifier able to accurately predict response (AUC: 0.80;5-fold cross validation). In contrast, Bychkov et al.^34^ showed that their HER2 status score was associated with survival hazard ratio on a trastuzumab-treated cohort. That approach, while conceptually informative, lacks the direct clinical applicability of a binary response predictor as we have presented in this study. Indeed, we showed that the HER2 status classifier is a weak predictor of trastuzumab response. In contrast the classifier trained on pre-treatment samples performs significantly better, demonstrating the value of directly predicting anti-HER2 response efficacy and suggesting the need for additional biomarkers to augment HER2 status for treatment recommendation.

In summary, the methodology that we have developed in this study provides an accurate and reproducible H&E-based approach for detection of HER2 status and response to trastuzumab therapy. Given that many new drugs have emerged for treatment of patients that express HER2, a combination of an AI classifier with conventional methods might improve the ability to select which HER2 drug is most likely to benefit each patient. Future prospective trials in the neoadjuvant setting are being considered. Furthermore, we anticipate that this approach will be generalizable to other cancer types and treatment outcome predictions. Identification and mapping of predictive features extracted by the CNN models on the H&E images can increase the interpretability of the results and aid in diagnostics. In future work, we plan to investigate the hierarchy of features extracted from H&E images for predicting HE2 status and response to trastuzumab.

## Data Availability

Data are available upon request.

## CODE AVAILABILITY

Codes are available upon request.
